# Interaction of KRSR Peptide with Titanium Dioxide Anatase (100) Surface: A Molecular Dynamics Simulation Study

**DOI:** 10.3390/ijms222413251

**Published:** 2021-12-09

**Authors:** Tamás Tarjányi, Ferenc Bogár, Janos Minarovits, Márió Gajdács, Zsolt Tóth

**Affiliations:** 1 Department of Oral Biology and Experimental Dental Research, Faculty of Dentistry, University of Szeged, Tisza Lajos Körút 64-66, H-6720 Szeged, Hungary; minimicrobi@hotmail.com (J.M.); gajdacs.mario@stoma.szote.u-szeged.hu (M.G.); 2 MTA-SZTE Biomimetic Systems Research Group, Eötvös Loránd Research Network (ELKH), Dóm tér 8, H-6720 Szeged, Hungary; bogar@sol.cc.u-szeged.hu; 3 Department of Experimental Physics, Faculty of Science and Informatics, University of Szeged, Dóm tér 9, H-6720 Szeged, Hungary; ztoth@physx.u-szeged.hu

**Keywords:** KRSR, peptide, titanium dioxide, osseointegration, REMD, umbrella sampling, PMF

## Abstract

Due to its tensile strength and excellent biocompatibility, titanium (Ti) is commonly used as an implant material in medicine and dentistry. The success of dental implants depends on the formation of a contact between the oxidized surface of Ti implant and the surrounding bone tissue. The adsorption of proteins and peptides to the implant surface allows the bone-forming osteoblast cells to adhere to such modified surfaces. Recently, it has been observed that tetrapeptide KRSR (Lys-Arg-Ser-Arg) functionalization could promote osteoblast adhesion to implant surfaces. This may facilitate the establishment of an efficient bone-to implant contact and improve implant stability during the healing process. GROMACS, a molecular dynamics software package was used to perform a 200 ns simulation of adsorption of the KRSR peptide to the TiO_2_ (anatase) surface in an aqueous environment. The molecule conformations were mapped with Replica Exchange Molecular Dynamics (REMD) simulations to assess the possible peptide conformations on the anatase surface, and the umbrella sampling method was used to calculate the binding energy of the most common conformation. The simulations have shown that the KRSR peptide migrates and attaches to the surface in a stable position. The dominant amino acid residue interacting with the TiO_2_ surface was the N-terminal charged lysine (K) residue. REMD indicated that there is a distinct conformation that is taken by the KRSR peptide. In this conformation the surface interacts only with the lysine residue while the ser (S) and arg (R) residues interact with water molecules farther from the surface. The binding free energy of the most common conformation of KRSR peptide to the anatase (100) surface was ΔG = −8.817 kcal/mol. Our result suggests that the N-terminal lysine residue plays an important role in the adhesion of KRSR to the TiO_2_ surface and may influence the osseointegration of dental implants.

## 1. Introduction

Due to its tensile strength, remarkable corrosion resistance, and excellent biocompatibility, titanium (Ti) and its alloys are commonly used as an implant material in medicine and dentistry [1,2,3,4]. When exposed to water or air, the surface of Ti is oxidized, and the titanium dioxide (TiO_2_) layer formed affects the interaction of Ti implants with various host tissues and cell types [5,6,7]. In the case of dental implants, the contact between the Ti implant and the surrounding bone tissue is of critical importance, especially in the context of osseointegration [8]. During the initial phase of osseointegration, proteins and peptides adsorb to and modify the implant surface [9]. It was demonstrated that functionalization of TiO_2_ surfaces with suitable peptides or proteins could enhance TiO_2_–bone interaction and promote the long-term survival of the implants [10,11]. In an in vivo rat tibia model of implantation, Ti pins coated by type I collagen or the tripeptide RGD (Arg-Gly-Asp; a motif present in several extracellular matrix proteins) attracted higher numbers of macrophages and osteoblasts, compared to unmodified Ti pins [12]. RGD is recognized by integrin proteins located at the membrane of various cell types. Integrin–RGD interactions facilitated cell-adhesion to the extracellular matrix, as well as to RGD-coated Ti surfaces [13,14,15]. Another peptide, KRSR (Lys-Arg-Ser-Arg) composed of four amino acids, is also suitable for modifying TiO_2_ surfaces. KRSR is a heparan-sulfate-binding peptide motif carried by different bone-adhesive proteins [16]. KRSR binds to cell surface proteoglycan molecules and promotes the adhesion of osteoblasts [17,18,19]. Similarly to their RGD-coated counterparts, KRSR-coated TiO_2_ surfaces show enhanced osteogenic differentiation [17,18,19].

The most common crystalline polymorphs of TiO_2_ are rutile, anatase, and brookite. The structure of rutile is tetragonal (a = b = 0.4584 nm, c = 0.2958 nm), anatase is also tetragonal; compared to rutile, however, the vertical axis of the anatase crystals is longer (a = b = 0.3782 nm, c = 0.9514 nm), brookite has an orthorombic structure (a = 0. 5436 nm, b = 0.9166 nm, c = 0.5135 nm) [20]. Ti surfaces are covered most commonly by mixtures of anatase and rutile [20]. The Ti implants, used in clinical practice, are treated by acids that favors forming of TiO_2_ surface with mainly anatase polymorphic properties [21]. The different surface characteristics of anatase and rutile may affect their interactions with biomolecules [6,11,22,23]. It was suggested that on the anatase (001) surface, the adsorption energy of important peptide motifs carried by bone-adhesive proteins is higher, compared to the rutile (110) surface, which may enhance the osseointegration of Ti implants covered by the anatase polymorph of TiO_2_ [22,24,25].

Based on molecular dynamics (MD) simulations, various models were generated describing the interactions of water molecules, amino acids and peptides, including RGD, with TiO_2_ surfaces [26,27,28,29,30,31,32,33]. Wu et al. used MD methods to simulate RGD peptide-TiO_2_ interaction with two water models (SPC/E and TIP3P) [26]. They used a similar system as our studied model. In their simulation they used rutile while we built up anatase. They found out that the RGD-surface interaction is sensitive to the initial arrangement of the peptide. Song et al. also used MD methods to simulate RGD on rutile surface, they have observed that the RGD adsorbs to the surface by guanidino, amino and carboxyl groups [27]. Wang et al. reviewed studies on MD and Density Functional Theory (DFT) methods for different biomolecule-biomaterial interactions [28]. They compared the hydroxyapatite (HA), TiO_2_, and graphene oxide (GO) surfaces and emphasized the importance of water environment in such studies [28]. Sowmiya and Senthilkumar used DFT methods to calculate the binding energy of proline, hydroxyproline and glycine on anatase (001) surface [29]. They found that these amino acids strongly bind to the anatase surface. In a follow-up study, the same authors have also calculated the RGD tripeptide anatase (001) adsorption energies [30]. Luan et al. used MD methods to simulate a nano amorphous TiO_2_ sphere particle with a biomolecule [32]. In our study, we utilized the same TiO_2_ Lennard-Jones parameters for Ti and O atoms in the TiO_2_ surface, as Luan et al. We simulated the anatase-KRSR interactions on molecular level using MD, for which—unlike for the RGD and implant interactions—no previous studies have been performed In vitro experiments demonstrated, however, that dual-coating of Ti surface with RGD and KRSR peptides enhanced synergistically the differentiation of a human osteoblastic cell line [34]. The aim of our MD simulations was to understand the KRSR behavior on the anatase surface in an aqueous environment and to calculate the binding energy of the predominant adsorption conformation of the peptide.

## 2. Results and Discussion

### 2.1. MD Simulation

**Figure 1:**
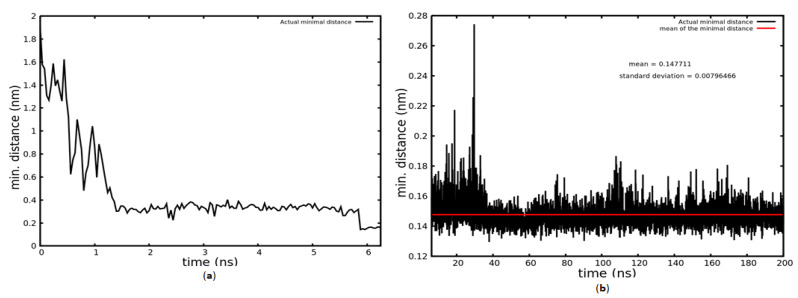
The distance between the TiO_2_ surface and the nearest peptide atom over time. In (**a**), the first 6.25 ns is shown; in (**b**), the last 193.75 ns is shown.

Firstly, a 200 ns MD simulation was performed. In this simulation the migration and attachment of a KRSR molecule onto the TiO_2_ anatase (100) surface in an aqueous environment was modelled. The interaction of water molecules with the TiO_2_ surface could already be observed during the first picoseconds of the simulation. The KRSR peptide–TiO_2_ surface interactions occurred in the first nanoseconds. As demonstrated on the left side of Figure 1a, the closest atom of the KRSR peptide was initially located at 1.9 nm from the TiO_2_ surface. As proposed for the adsorption of peptides to solid surfaces [35,36], the binding of the KRSR peptide to the anatase 100 surface also occurred in three subsequent phases during the simulation. In the first phase of adsorption, which lasted about 1.5 ns (see Figure 1a), the KRSR peptide moved from the bulk water toward the anatase, approaching the water–TiO_2_ interface. In Supplementary Material Video S1, this migration phase can be seen in video form. At 1.5 ns, the minimal distance between the peptide and surface was close to 0.2 nm due to non-bonded (van-der Waals and Coulomb) interactions. After 6 ns, the mean minimal distance between the KRSR peptide and the TiO_2_ surface fell below 0.2 nm, possibly due to anchoring of the peptide to the surface during the second phase of adsorption (Figure 1a).

The KRSR–TiO_2_ surface interaction was stable until the end of the simulation. In the last 193 ns, the closest atom of the peptide to the surface oscillated with a 0.02 nm amplitude, at 0.147 nm average distance from the surface. This third phase of adsorption could correspond to lockdown of the peptide to the surface (Figure 1b). Note that, in Figure 1b, a reduced distance scale is used compared to Figure 1a.

**Figure 2:**
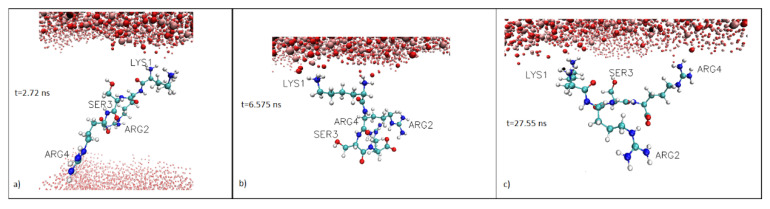
Snapshots from the simulation. (**a**) time t = 2.72 ns (**b**) time t = 8 ns; (**c**) t = 27.55 ns. The snapshots were made without the water molecules. Color code: oxygen, red; nitrogen, dark blue; carbon, cyan; titanium, ochre; hydrogen, gray. Visualization was done with VMD 1.9.3 in CPK representation.

The 200 ns simulation showed that the KRSR peptide approached the TiO_2_ surface and after 1 ns simulation time it interacted with the surface, mainly with the charged N-terminal lysine residue (see LYS1, Figure 2a). During the second adsorption phase (t > 6 ns) the dominant interaction also occurred via the N-terminal lysine residue, as shown on the snapshot in Figure 2b.

We noticed that, in addition to the initial interaction involving the lysine residue, the charged arginine residues (ARG2 and ARG4) also contacted the TiO_2_ surface, although less frequently. Figure 2c demonstrates an example of ARG4 and LYS1 contacting the surface simultaneously.

We calculated the Root Mean Square Deviation (RMSD) values of the KRSR peptide over time. The RMSD is the variation of atomic positions compared to the initial structure of the KRSR peptide during the 200 ns simulation. The calculated values indicate that the atomic coordinates of the peptide chain do not change drastically during the simulation. The mean value of the RMSD was 0.116 nm and its standard deviation is 0.016 nm.

### 2.2. Determination of Adhered Peptide Conformations by Replica Exchange Molecular Dynamic Simulation (REMD)

**Figure 3:**
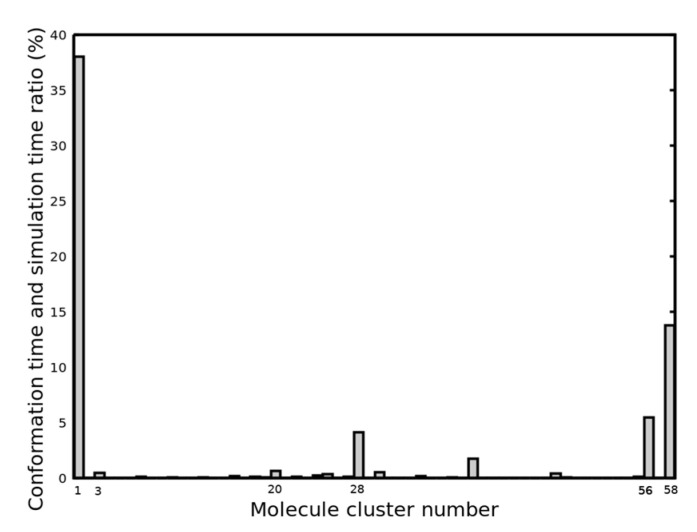
Cluster populations of the different KRSR peptide conformations, based on the lowest temperature simulation.

REMD simulations were performed on KRSR to investigate the conformational behavior of the peptide at different temperatures. A total of 96 replicas were used in the temperature range of 310–454 K, and the final analysis was performed for the replica at 310 K. The linkage clustering method was used, with a 0.1 nm RMSD cutoff to determine different molecular conformations of the KRSR on the surface. Conformational analysis of the KRSR provided insight into the unstable and disordered structures of the KRSR. Figure 3 shows the distribution of the occurrence of peptide molecule conformations for the first 58 clusters, which were the most populated. The different molecular cluster conformations were determined by the RMSD.

**Figure 4:**
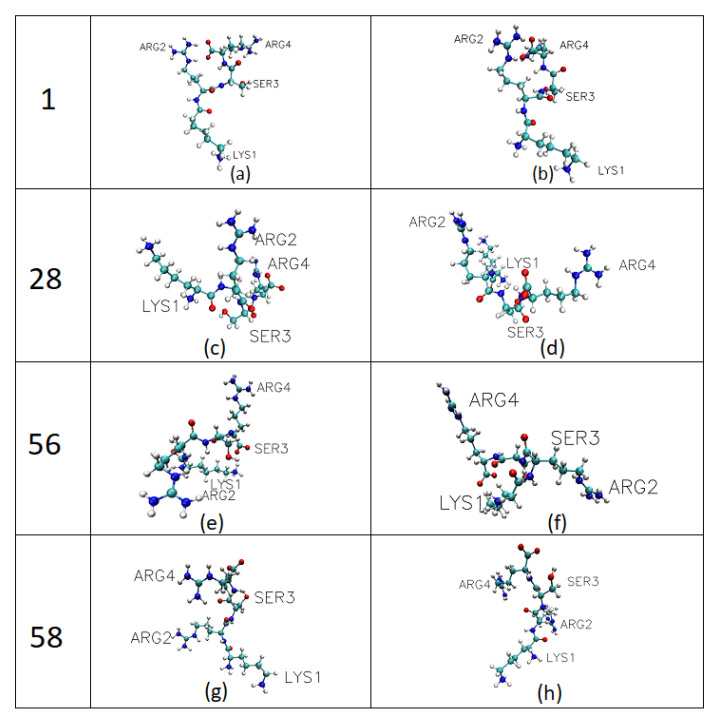
Molecular conformations of the KRSR peptide viewed from two different angles. The four most prevalent conformations identified by REMD simulations are shown. (**a**,**b**) are views from different directions of KRSR conformation cluster number 1, (**c**,**d**) are views of cluster number 28, (**e**,**f**) shows the conformation of cluster number 56 and (**g**,**h**) belongs to conformation of cluster number 58. Visualization was done with VMD 1.9.3 in CPK representation.

The four most prevalent KRSR peptide conformations identified by the clustering process corresponded to cluster number 1 (cluster size: 4751), 28 (cluster size: 516), 56 (cluster size: 685), and 58 (cluster size: 1724), respectively; their molecular geometries are displayed in Figure 4. In total, 468 clusters were found and 4539 transitions were recorded. All four conformations contacted the surface through the N-terminal lysine residue (Lys1), however, conformation 56 and 28 also adhered with the second residue (Arg2).

### 2.3. Binding Free Energy Calculation by Potential of Mean Force (PMF) Calculation

**Figure 5:**
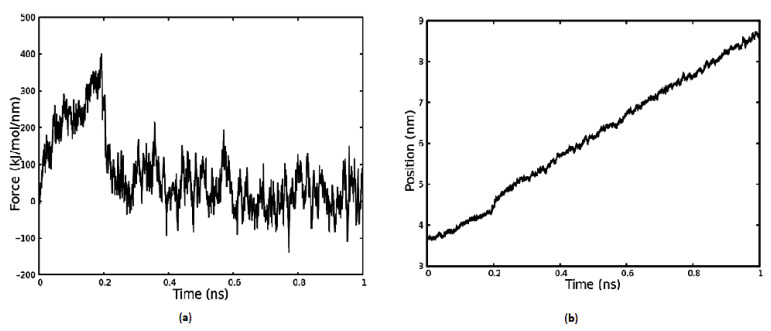
In (**a**), the force that is necessary to pull the KRSR peptide’s center of mass is represented as a function of time. In (**b**), the z coordinate of the peptide’s center of mass is depicted as a function of time. The surface of the TiO_2_ slab is between 0 and 3.5 nm.

Based on the results obtained from REMD simulations, we used the most dominant conformation to pull it from the surface. The magnitude of the force required for pulling the KRSR molecule (cluster No.: 1) is shown in Figure 5. This is done by attaching an imaginary spring to the center of mass (COM) of the molecule, which is pulled along the z-axis perpendicular to the TiO_2_ surface. The spring constant was defined as 1000 kJ∙nm^−1^∙mol^−1^, which is the usual value for pull procedures. The rate of pulling was set to a constant value of 5 nm/ns, which is comparable to the velocity of molecular motion in fluids. During the pull-simulation, interactions occur (i) between the anatase surface and the peptide, and (ii) between the surrounding water molecules and the peptide. The pulling procedure lasted up to 1 ns. During pulling, up to 0.2 ns, the center of the molecule moves away from the surface, which can be seen in the Supplementary Material Video S2. The molecule elongates and at 0.2 ns the peptide-TiO_2_ surface interaction ceases, and the molecule then has close contacts only with the surrounding water molecules. In Figure 5a, at 0.2 ns this is accompanied by a sudden decrease in force. In Figure 5b, the Z position of the center of mass of KRSR molecule is shown as a function of time. The position value starts from 3.8 nm since the surface Z position ends roughly at this value. During the elongation period the position value gradually increases and at 0.2 ns a sudden sub nm increase can be observed, which is in correlation with the force–time curve. This sudden increase refers to that moment, when the elongated peptide is torn from the surface and contracts. After 0.2 ns, the position of the peptide generally corresponds to the 5 nm/ns pulling velocity, and the interactions with the water surrounding can be seen as the noise on the curve.

**Figure 6:**
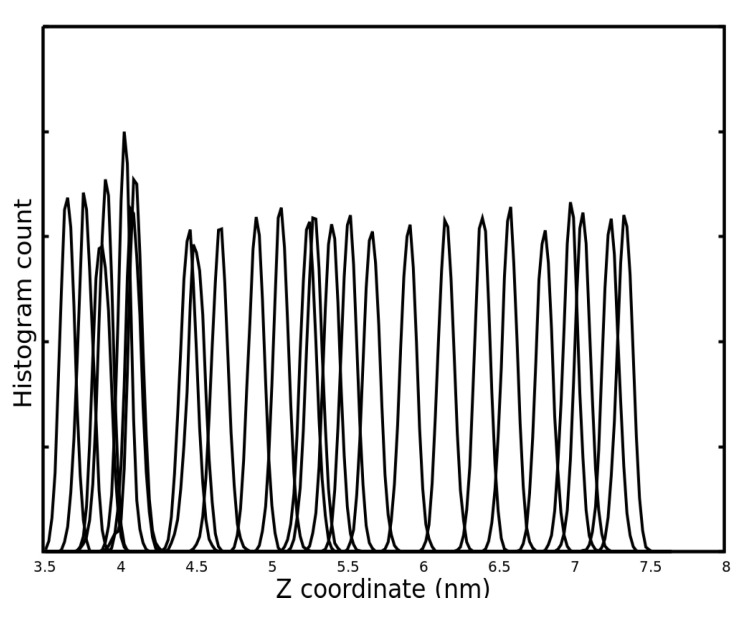
Distribution of the distances taken by the KRSR peptide’s center of mass from the TiO_2_ surface at different fixed points during the 10 ns sampling.

During the pull simulation we recorded the atomic positions of the system for the umbrella sampling procedure. The center of mass of the peptide was fixed in 24 different positions with a harmonic potential at different points along the pull axis. These points were taken further away from one after the other. We ran independent molecular dynamics simulations with these fixed KRSR systems where the position of COM of the peptide could be displaced due to the interaction with water molecules. The distribution of the displacements gives an umbrella-type curve at each position. The results of the umbrella sampling are shown in Figure 6 in a histogram form. This histogram displays the distribution of the distances taken by the COM of the KRSR peptide from the TiO_2_ surface at the 24 fixed positions described above, during the 10 ns sampling.

**Figure 7:**
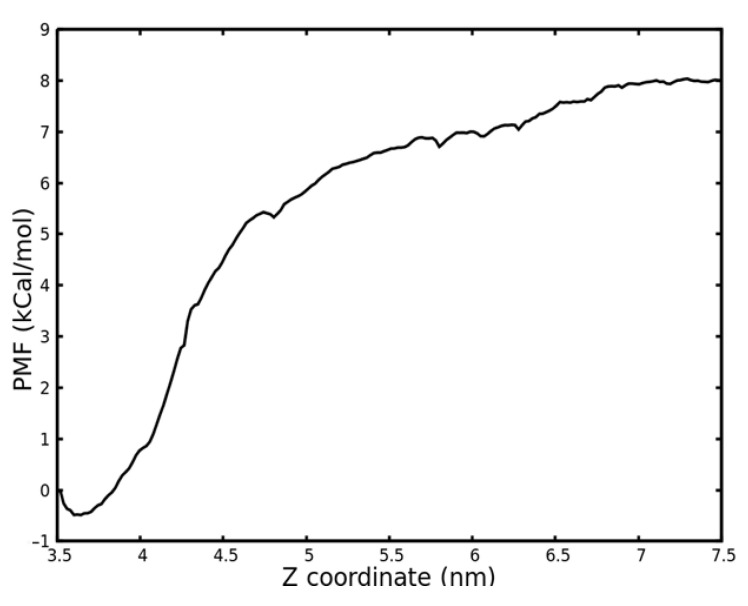
Potential of Mean Force (PMF) energy values of the simulated KRSR peptide–TiO_2_ surface interaction as a function of the Z coordinate.

Finally, the Weighted Histrogram Analysis Method (WHAM) algorithm [37] was used to analyze the results of the umbrella sampling method. As a result, we obtained the potential of mean force (PMF) curve shown in Figure 7. The highest and lowest values correspond to the ΔG value of the bonding/release process. The binding free energy ΔG was established, based on the PMF profile shown. Thus, ΔG, the binding free energy of the KRSR peptide to the anatase (100) surface obtained, was −8.817 kcal/mol (−36.89 kJ/mol).

### 2.4. Discussion

Biomolecules bound to the surface of Ti implants covered by TiO_2_ may facilitate successful integration of both surgical and dental implants into the surrounding bone tissue [11,38,39]. It was demonstrated that distinct proteins and peptides attached to TiO_2_ surfaces enhance migration, proliferation, and differentation of osteoblasts, which may play a prominent role in bone formation [12,17,23,34,40,41]. In addition to in vivo and in vitro studies, molecular dynamics simulations are also indispensable for the understanding of the complexities of peptide–water–solid surface interactions involved in osseointegration of implants [22,35,42,43,44].

We have performed MD simulations to study the adsorption of the KRSR peptide to the anatase (100) surface in aqueous environment. Three phases of KRSR binding were observed. In the initial phase (Phase 1), lasting for 1.5 ns, the peptide moved from the bulk water toward the anatase surface. This corresponds to a migration speed of ~1.14 m/s. In Phase 2, from 1.5 ns to 6 ns, the peptide was anchored at about 0.2 nm from the surface. Firstly the lysine (K) part of the peptide interacted with the surface. This interaction led to anchoring of the peptide to the surface, which showed stable binding in the second and third phase of adsorption. In Phase 3 (lockdown; from 6ns to 200 ns), the KRSR peptide stayed stably in the vicinity of the anatase 100 surface: its closest atom was located at an average of 0.147 nm from the surface till the end of the simulation. Our prolonged, 200 ns simulation revealed that the initial KRSR-TiO_2_ (anatase 100) surface interaction was mediated by the N-terminal lysine (K) residue of the peptide. It was also observed that, in certain time periods, the two charged arginine (R) residues, Arg2, which is next to the Lys1, and the carboxy terminal Arg4 residue also interacted with the surface. The fluctuations of the peptide geometry, having an average amplitude of 0.02 nm, can be attributed to the dynamic interactions between the peptide, surface, and water molecules. These observations are compatible with the results of Sultan et al., who applied molecular dynamics simulations and metadynamics to calculate the binding affinities of distinct amino acid analogues to the charged, aqueous rutile (110) surface [45]. They found that at neutral pH the ranking order of the strongest binder side chain analogues was ”arginine”, ”lysine”, ”aspartic acid”, and ”serine” [45].

**Figure 8:**
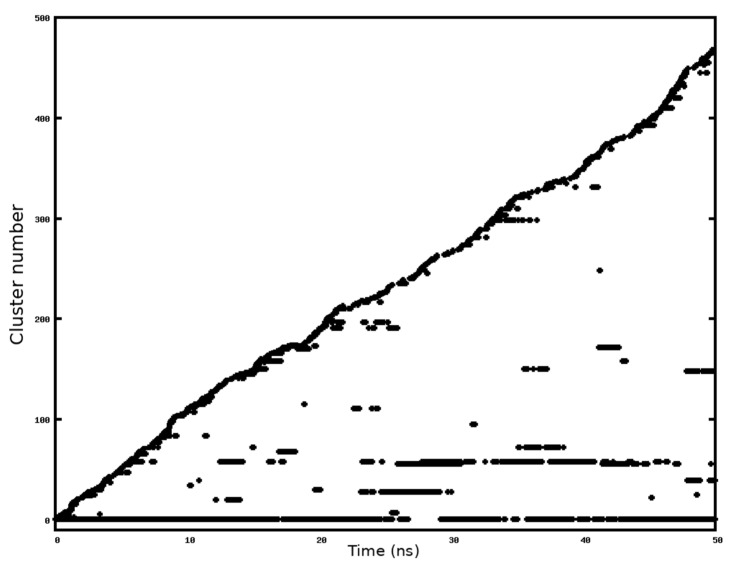
Changes in cluster populations of the different KRSR peptide conformations during the REMD simulation.

We assessed the most probable molecular conformations of the KRSR peptide using REMD (Replica Exchange Molecular Dynamics) simulations and selected the most likely conformation to calculate the binding energy characteristic for the KRSR-anatase (100) interaction. Figure 3 shows that cluster 1 appears in almost 40% of simulation times, as shown in Figure 8 as well.

Figure 8 demonstrates how the most prevalent molecular configurations (clusters) change over the REMD simulation. Although cluster 1 was present from the beginning of the simulation, new molecular conformations also occurred due to the interactions with the TiO_2_ surface and water molecules. Furthermore, during the higher temperature simulations the molecule coordinates can replica change with a certain probability. It is also visible that the KRSR peptide during this simulation reverts to more favorable conformations (to the previously discussed four dominant conformations shown in Figure 4).

Out of the four most common conformations, No. 1 and 58, (No. 58 is the second most common manifestation) are similar in that only the N-terminal lysine resiude (Lys1) adheres to the surface. However, at conformations 56 and 28, arginine (Arg2) also adheres in addition to Lys1, forming a bridge; whereas Ser3 and Arg4 residues interacts with the surrounding water molecules farther away from the surface.

Pulling of the KRSR molecule in aqueous environment and the umbrella sampling procedure were used to calculate the binding free energy of the KRSR to the anatase 100 surface. Figure 5b and Figure 6 shows the detachment of the molecule from the surface when applying an elastic force that is maintained by a harmonic potential. In Figure 5b, a sudden change was observed at 0.2 ns time point indicating the detachment of the molecule from the TiO_2_ surface. As demonstrated in Figure 6, the distributions of the distances (normal distributions) at different time points are evenly dispersed along the Z coordinates. However, when the center of mass of the KRSR molecule is between 4 and 4.5 nm, a rarefaction can be observed. Both curves indicate the disintegration of the peptide from the surface. Our main result is the determination of the ΔG, the binding free energy of the KRSR peptide to the anatase (100) surface for which the −8.817 kcal/mol (−36.89 kJ/mol) value was obtained. This value is in the expected range compared to the results of other TiO_2_–molecule binding energy studies.

Luan et al. analyzed the interaction of the SH3 domain (SRC-homology 3 domain, derived from an adaptor protein) with a TiO_2_ amorphous nanoparticle [32]. In their study they verified the very same Lennard-Jones potential parameters of Ti and O as used in this work. Brandt and Lyubartsev et al. applied the umbrella sampling method to calculate the free energy profiles and the adsorption free energies of 19 amino acids on the rutile (100) TiO_2_ surface [46]. It is worth noting, however, that the polar side chain analogues of the amino acids serine (Ser, S) and threonine (Thr, T) were able to penetrate the tightly-bound water layer on the TiO_2_ (rutile, 100) surface and bind to TiO_2_ [46]. Arcangeli et al. used DFT and MD methods to see the structure and stability of single amino acids; they also used MD methods to investigate the adsorption of the 13-residue sequence AMRKLPDAPGMHC by pulling it away from the surface [47]. They used a method similar to the one we used to pull the KRSR peptide from the surface in our study. Previously Lemkul et al. used the umbrella sampling and pulling method to calculate binding free energies protofibrils [48]. In their study they pulled away peptide parts, this is also very similar how we performed our pulling method and umbrella sampling.

Muir et al. used DFT methods to investigate the RGD binding energy on the TiO_2_ rutile (110) surface [49]. Their data indicated that the peptide adsorps via the carboxyl group of the aspartic acid residue to the surface. In their study it was found that the RGD O atom made a sigma bond with the surface Ti atom. Sowmiya et al. also used DFT methods to investigate the adsorption of RGD but on an anatase TiO_2_ (001) surface [30]. They found that the adsorption energy for RGD is higher on anatase than on the rutile surface and concluded that the anatase surface is more favorable for implant functionalization. The maximum adsorption energy in their study for RGD on the anatase surface was −1.62 eV (−6.2 kcal/mol = −25.94 kJ/mol), which is consistent with our result, KRSR anatase adsorption energy (−8.817 kcal/mol). Thus, although the adsorption energies were determined by different methods, the results were, in a way, comparable for these charged oligopeptides.

As far as we know, this is the first attempt to determine the binding energy of the KRSR peptide to TiO_2_ (anatase) surface. Regarding peptide–TiO_2_ surface interactions, most of the molecular dynamics studies and DFT calculations focused on the binding of RGD peptide to TiO_2_ polymorphs, rutile and anatase. The RGD sequence is present in several extracellular matrix (ECM) proteins and mediates ECM–cellular receptor interactions [13,14,15]. Zhang et al. calculated that the binding energy of RGD is higher for the anatase (101) surface than to the rutile (110) surface [22]. They argued that the presence of Ti atoms on the surface of the anatase positively affects the surface adsorption of the RGD peptide, while on the oxygen-terminated rutile surface the interaction with oxygen atoms is weaker [22]. In addition, comparison of the RGD binding energy in vacuum and water revealed that the presence of water molecules may interfere with the adsorption of the peptide to TiO_2_ surfaces [22]. We have also observed that water affects the binding of KRSR, and the binding of the ε-amino group of the N-terminal lysine occurs mostly between the oxygen atom of the TiO_2_ surface or the surface-bound water. The results of Schneider et al. are consistent with this observation, they found that RGD binding to the TiO_2_ surface is mediated predominantly by the arginine (R) residue located to the N-terminal end of the peptide [50]. They suggested, however, that the C-terminal asparagine (D) also plays a role in the surface association as well, in accordance with the proposal by Guo et al. [50,51]. It has to be noted that the integrin-binding RGD motif interacted with a subset of integrin family receptors expressed on the surface of a variety of cell types [52]. In contrast, the heparin-binding tetrapeptide KRSR preferentially stimulated the adhesion of osteoblasts to KRSR-coated surfaces [17,18,19,34]. We observed that during molecular simulation the KRSR peptide attached to the TiO_2_ (anatase) surface predominantly via its N-terminal lysine (K) residue, whereas the serine (S) and arginine (R) residues interacted with water molecules. This observation suggests that the KRSR motifs carried by certain extracellular matrix proteins may also interact with titanium surfaces via the side-chain amino group of lysine residues and may also facilitate the design of biomimetic surfaces enhancing osseointegration of dental or surgical implants.

## 3. Materials and Methods

**Figure 9:**
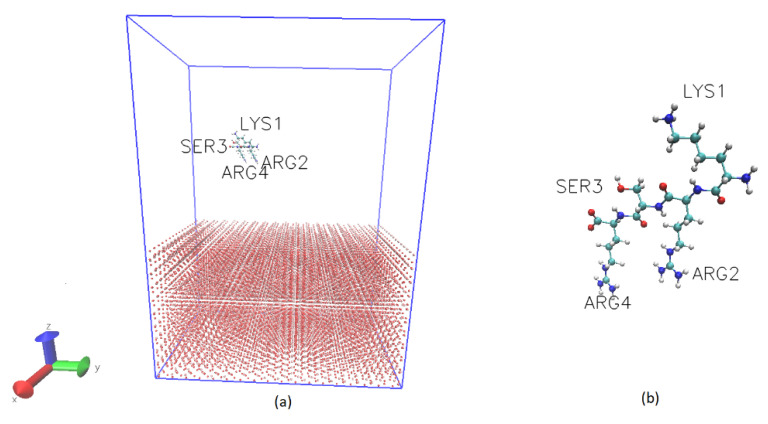
Snapshots of the initial geometry of the simulated system: (**a**) The left snapshot shows the TiO_2_ crystal structure and the KRSR peptide, without the water molecules, the blue box represents the periodic boundary; (**b**) The right snapshot shows the initial KRSR peptide geometry. Color code: oxygen, red; nitrogen, dark blue; carbon, cyan; titanium, ochre; hydrogen, gray. Visualization was done with VMD 1.9.3 in CPK representation.

Molecular dynamics (MD) simulations were performed with GROMACS (2018.3 version) [53,54,55,56,57,58,59,60]. The CHARMM36 force field [61,62] was applied to set up the parameter files for the KRSR and its surrounding environment. This force field is suitable for generating polypeptide backbone conformational ensembles for intrinsically disordered peptides and proteins. The simulation space was a 4.5 nm × 8.3 nm × 11 nm box. This space was convenient to define the TiO_2_ crystal lattice. The space above the Ti surface was filled with the peptide, water, and a few ions (Na^+^ and Cl^−^) to maintain the neutrality. The TiO_2_ crystal structure (100) started from z = 0 nm until z = 4 nm. The simulation box was set to have a periodic boundary condition in order to simulate a large space. This was necessary to make the peptide and the molecules in the solution able to migrate over the boundary. For the setting up of the TiO_2_ crystal, the unit cell of anatase was used. The structure of unit cell was obtained from the American Mineralogist Crystal Structure Database recorded by Howard [63]. The KRSR peptide initial geometry was created with the program Gabedit 2.5.0 [64]. After the creation process, an energy minimization was performed on the peptide with the Quasi Newton gradient method (Amber potential). At the beginning of the molecular dynamics simulation, the position of the KRSR peptide was set to the middle of the x-y plain and ~1.9 nm distance from the anatase surface. The simulation system contained 7625 water molecules and the TIP3P explicit water model [65] was applied on them. The initial geometry of the system is shown in Figure 9a.

During the simulation, a cut off distance of 1 nm was applied for the electrostatic and van der Waals forces. The van der Waals potential for TiO_2_ was described with the usual Lennard-Jones potential parameters as described by Luan et al. [32] (Equation (1)):

(1) $$U\left(r_{ij}\right)=\varepsilon_{ij}\left[{\left(\frac{\sigma_{ij}}{r_{ij}}\right)}^{12}-2{\left(\frac{\sigma_{ij}}{r_{ij}}\right)}^{6}\right]+\frac{q_{i}q_{j}}{r_{ij}}$$

where the first part in Equation (1) is the Lennard-Jones potential and the second part is the Coulomb potential, *r_ij_* is the distance between two atoms, *ε_ij_* is the well depth of the potential energy, *σ_ij_* is the size of the particles, *q_i_* is partial charges.

The following parameters were used to set the Lennard-Jones potential parameters for the Ti and O atoms in the anatase crystal: ε_Ti−Ti_ = 0.58 kcal/mol, ε_Ti−O_ = 0.424 kcal/mol, ε_O−O_ = 0.58 kcal/mol, σ_Ti−Ti_ = 0.220 nm, σ_Ti−O_ = 0.272 nm, σ_O−O_ = 0.324 nm and the partial charges q_Ti_ = 2.196 e, q_O_ = −1.098 e. The potential parameters for the atoms in the solution were already defined by the CHARMM36 force field. The simulation protocol started with a usual energy minimization in 2000 steps to relax the initial structure. After this, the temperature of the system was increased from 0 K up to 310 K and the equilibration was done in two phases. During the first phase an isothermal-isochoric (Number of particles, Volume, and Temperature are all constant: NVT) process was done over 500 ps. The system was heated up in two different groups: (i) atoms of the anatase crystal and (ii) the peptide–water components. These two groups of atoms were treated like this during all simulation processes. The temperature was controlled via Langevin dynamics [66]. The equilibrium searching process continued with the isothermal-isobaric equilibration (Number of particles, Pressure and Temperature are all constant: NPT) during another 100 ps. A 200 ns MD simulation started after this point at 310 K and 1 bar pressure. The time step during the NVT and NPT equilibration processes were 1 fs, while it was 2 fs during the MD. During the simulations there was no extra restrictions for the TiO_2_ atoms. The atoms stayed near at their crystal lattice points and vibrated there with the given kinetic energy.

For REMD (Replica Exchange Molecular Dynamics) simulations, 96 replicas were used to find the incidence of different conformations of the KRSR molecule. The temperature difference was 1.5 K, starting at 310 K on the lowest temperature thread. The initial system for the REMD was taken from the result of the 200 ns MD simulation. The replicas were equilibrated (NPT) over 1.25 ns. After the equilibration we performed a 50 ns parallel simulation on the replicas. During the equilibration and REMD simulations the time step was 2 fs. The replica exchange could take place at each 500th step, i.e., at 1 ps intervals. The replica exchange only replaced the atomic coordinates of the peptide by adjacent temperature simulations. The probability of replacements throughout the simulation was around 0.3. By examining the lowest temperature thread, the prevalence of distinct peptide conformations was assessed with a 0.1 nm cut-off single linkage clustering.

The Potential Mean Force (PMF) simulations were performed using conformation 1, the most abundant conformation observed during REMD (Figure 4a). During the PMF calculations the simulation box was extended in the z axis to 18 nm. The size of the new, extended simulation box was 4.5 nm × 8.3 nm × 18 nm. As a first step, the center of mass of the KRSR peptide was pulled from the TiO_2_ surface to a distance of 5 nm, over 1 ns. The z coordinate represents the COM coordinate of KRSR at the z axis, which is perpendicular to the plane of the anatase–water interface. After this process at 24 selected points the COM of KRSR position was fixed with a harmonic force. At these points, umbrella sampling was performed with a potential energy well depth of 1000 kJ·mol^−1^·nm^−2^, as described in the literature [48,67]. The equilibrium state search lasted 1.2 ns, after this a 10 ns MD simulation was performed with a 2 fs time step. To create the PMF profile the WHAM (weighted histogram analysis method) module of GROMACS was applied on the 24 selected umbrella samples [34]. Based on our umbrella sampling simulations, the binding energy of the KRSR peptide to the surface of the anatase was determined.

## Data Availability

External data sources used in this study are cited in article. All data generated during the study are presented in this paper and its supplementary materials.

## Supplementary Materials

The following are available online at https://www.mdpi.com/article/10.3390/ijms222413251/s1.
